# Mass Spectrometry Imaging‐Assisted Discovery of Gallotannin Biosynthetic Genes in the Root of *Paeonia suffruticosa*

**DOI:** 10.1002/advs.202514010

**Published:** 2026-03-15

**Authors:** Yushi Liu, Wenna Duan, Weiwei Tang, Yucheng Zhao, Bin Li

**Affiliations:** ^1^ State Key Laboratory of Natural Medicines and School of Traditional Chinese Pharmacy China Pharmaceutical University Nanjing China; ^2^ Department of Resources Science of Traditional Chinese Medicines School of Traditional Chinese Pharmacy China Pharmaceutical University Nanjing China

**Keywords:** serine carboxypeptidase‐like gene, enzyme catalysis, gallotannins, glycosyltransferase gene, MALDI MSI, *Paeonia suffruticosa*

## Abstract

A highly efficient gene screening strategy for discovering enzymes responsible for gallotannin biosynthesis in the root of *Paeonia suffruticosa* was developed by integrating matrix‐assisted laser desorption/ionization mass spectrometry imaging (MALDI**‐**MSI), spatial transcriptome sequencing, and phylogenetic analysis. Through this strategy, one UGT gene (*PsUGT84A*) and eight serine carboxypeptidase‐like (SCPL) genes (*PsSCPL311*, *PsSCPL272*, *PsSCPL155*, *PsSCPL406*, *PsSCPL886*, *PsSCPL531*, *PsSCPL799*, and *PsSCPL979*) were first identified and characterized. The key UDP‐glycosyltransferase (UGT) PsUGT84A involved in the initial step of gallotannin biosynthesis can catalyze the synthesis of *β*‐galloylglucose (*β*G) from gallic acid. Subsequently, a series of SCPL acyltransferases enable the sequential acylation of the 6‐OH, 2‐OH, 3‐OH, and 4‐OH at the central glucose molecule of *β*G to ultimately synthesize the fully galloylated derivative, pentagalloylglucose (PGG). Furthermore, heterologous reconstruction of the gallotannin biosynthetic pathway in *Nicotiana benthamiana* was achieved. This study represents the first comprehensive elucidation of gallotannin biosynthesis in *P. suffruticosa*, providing essential enzymatic tools for the efficient production of hydrolysable tannins.

## Graphical abstract

An integrated approach combining mass spectrometry imaging, transcriptomics, and phylogenetic analysis facilitated the efficient identification of genes involved in gallotannin biosynthesis in Paeonia suffruticosa. As a result, a key UGT and several SCPL acyltransferases responsible for biosynthesizing pentagalloylglucose were successfully identified.

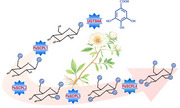

## 1 Introduction

Elucidating the biosynthetic mechanisms of plant secondary metabolites remains pivotal for unraveling plant biological characteristics and developing novel therapeutics. *Paeonia suffruticosa* Andr., a perennial deciduous shrub belonging to the family Ranunculaceae and genus *Paeonia*, has long attracted considerable attention from horticulturists and pharmacognosists due to its economic and medicinal significance [1, 2]. Modern pharmacological studies have confirmed that *P. suffruticosa* exhibits diverse bioactivities, including antioxidant, anti‐inflammatory, cardioprotective, anticancer, and antidiabetic effects [3], and provides valuable resources for drug discovery and advancements in the healthcare industry. Hydrolysable tannins represent one of the principal bioactive constituents isolated from *P. suffruticosa*. These compounds not only protect plants against biotic stresses [4] but also exhibit diverse pharmacological properties, including antioxidant [5], antidiabetic [6], antimicrobial [7], and antiviral activities [8], leading to their broad applications in the food, pharmaceutical, and animal husbandry industries [9, 10]. According to their core structural frameworks, hydrolysable tannins are classified into two categories: gallotannins (GTs) and ellagitannins (ETs). GTs feature a *β*‐D‐glucose core esterified with galloyl moieties, forming derivatives with varying degrees of gallic acylation. In contrast, ETs arise from the oxidative coupling of galloyl moieties in GTs to generate hexahydroxydiphenoyl units [11]. Notably, 1,2,3,4,6‐pentagalloylglucose (PGG) serves as both a key biosynthetic intermediate for GTs and a precursor molecule for ETs. Therefore, deciphering the biosynthetic machinery underlying PGG formation constitutes a central scientific challenge in elucidating the structural diversity of hydrolysable tannins in *P. suffruticosa*.

**FIGURE 1:**
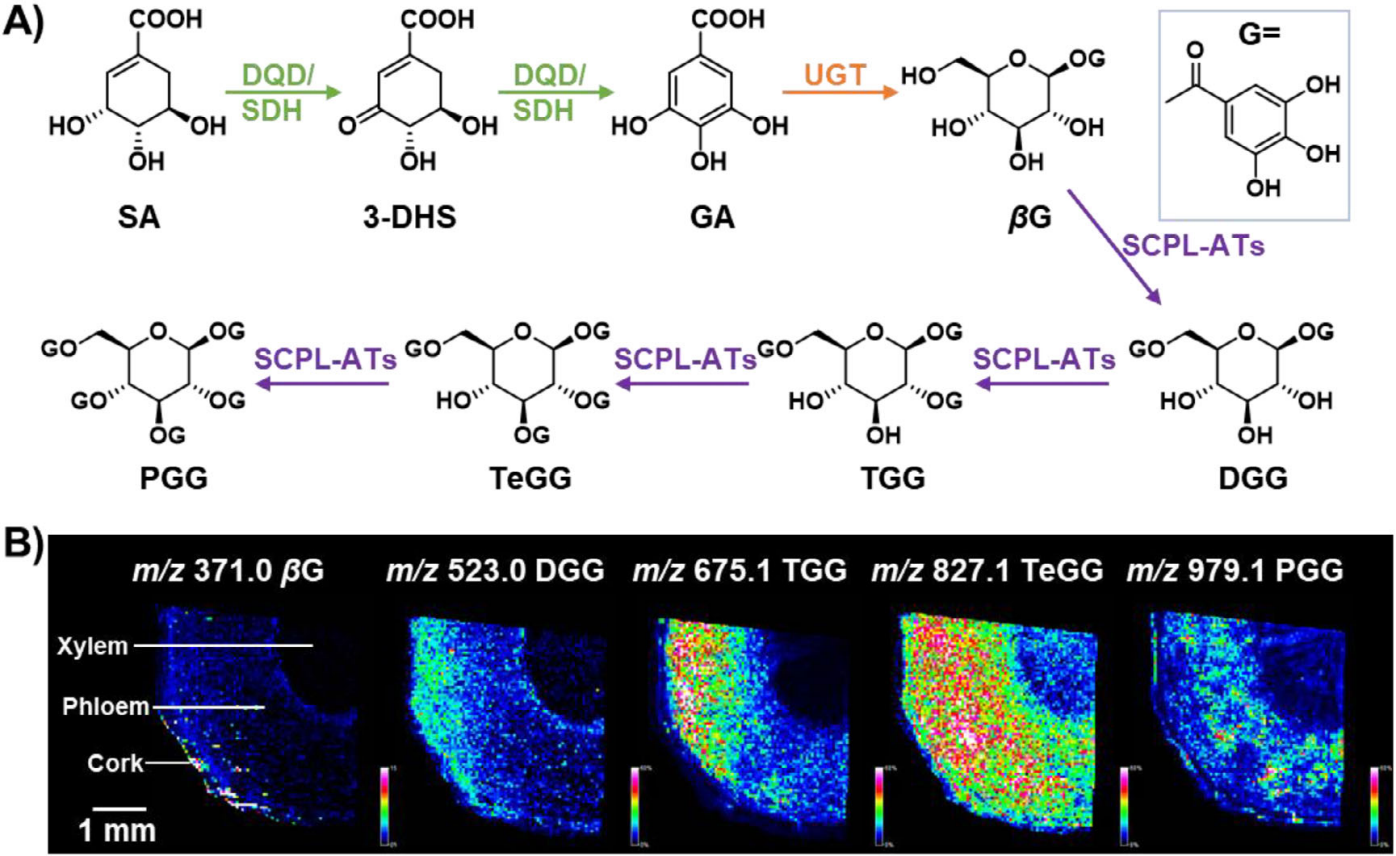
Proposed biosynthetic pathway of PGG and the spatial distribution of *β*G, DGG, TGG, TeGG, and PGG in *P. suffruticosa* root. (A) The proposed biosynthetic pathway of gallotannin and catalytic enzymes. DQD/SDH: dihydroorotate dehydratase/shikimate dehydrogenase, UGT: uridine diphosphate‐dependent glucosyltransferase, SCPL‐Ats: serine carboxypeptidase‐like acyltransferases. (B) MALDI‐MS images of selected gallotannin ions, including [*β*G+K]^+^ (*m/z* 371.0), [DGG+K]^+^ (*m/z* 523.0), [TGG+K]^+^ (*m/z* 675.1), [TeGG+K]^+^ (*m/z* 827.1), and [PGG+K]^+^ (*m/z* 979.1).

The biosynthetic precursor of gallotannins, gallic acid (GA), originates from the shikimate pathway. The initial upstream synthesis is relatively conserved across plants, where 3‐dehydroshikimate (3‐DHS), an intermediate of the shikimate pathway, undergoes dehydrogenation catalyzed by 3‐dehydroquinate dehydratase/shikimate dehydrogenase (DQD/SDH) to yield GA [12, 13]. Subsequently, GA is conjugated with UDP‐glucose via uridine diphosphate‐glycosyltransferases (UGTs) to form *β*‐glucogallin (*β*G), and the key genes for this reaction have been identified in *Quercus robur*, *Camellia sinensis*, and other plants (e.g., *QrUGT84A13* [14] and *CsUGT84A22* [15]). In subsequent gallotannin biosynthesis, serine carboxypeptidase‐like acyltransferases (SCPL‐ATs) catalyze the transfer of galloyl moieties to hydroxyl groups of the *β*G glucose core [16]. Intriguingly, hydroxyl substitution is not random but exhibits strict positional specificity. Early studies employing protein purification from plants and enzymatic assays revealed the role of *β*G in gallotannin biosynthesis, which served as the acyl donor involved in the sequential esterification occurring at the 6‐OH, 2‐OH, 3‐OH, and 4‐OH of the glucose core, ultimately generating the fully galloylated derivative PGG (Figure 1A). However, the genes encoding these activities remained unidentified [17, 18, 19, 20]. To date, only *CoSCPL3* from *Camellia oleifera* and *FaSCPL3* from *Fragaria ananassa* have been reported to catalyze four consecutive steps in hydrolysable tannin biosynthesis, leading to the formation of PGG [21, 22]. Whether *P. suffruticosa* harbors SCPL‐ATs with analogous functions or adheres to established catalytic paradigms for assembly of tannin remains uncharacterized.

Although genomic technologies have accelerated the investigation of natural product biosynthetic pathways, as exemplified by landmark studies on *Taxus* spp. and paclitaxel [23], *Leonurus japonicus* and leonurine [24], and *Salvia miltiorrhiza* and tanshinones [25], the targeted discovery of functional genes remains a significant challenge. The accumulation of plant secondary metabolites represents integrated outcomes of gene expression, protein function, and environmental interactions. Reliance on single‐omics approaches (e.g., transcriptomics) often fails to capture the dynamic correlations between the spatial distribution of metabolites and gene expression. In recent years, matrix‐assisted laser desorption/ionization mass spectrometry imaging (MALDI‐MSI) has emerged as a powerful tool for visualizing spatial distribution patterns of metabolites due to its high sensitivity and spatial resolution, providing critical insights into decoding natural product biosynthesis [26, 27]. Nevertheless, how to leverage MALDI MSI‐derived spatial clues with gene‐metabolite correlation networks remains an underexplored frontier. Therefore, an urgent need is for the establishment of an efficient strategy to correlate spatial metabolite distribution with their biosynthetic genes.

In this work, by integrating MALDI‐MSI, tissue‐specific transcriptomics, and phylogenetic analysis, the phloem of *P. suffruticosa* root, a major site responsible for PGG synthesis, was unraveled. Consequently, the UGT84A subfamily genes and homologous SCPL‐AT genes involved in the biosynthesis of gallotannins were discovered, and their function was verified for the first time, including one *PsUGT84A* and eight *PsSCPLs*. Furthermore, the active genes derived from *P. suffruticosa* were successfully heterologously expressed in the leaves of *Nicotiana benthamiana*, achieving de novo synthesis of PGG. This work not only confirms the effectiveness of the integration of multi‐omics data but also provides a generalizable framework for functional gene discovery.

## 2 Results and Discussion

### 2.1 Spatially Resolved Analysis of the Tissue Distribution Patterns of Gallotannins in *P. suffruticosa* Root by MALDI‐MSI

*P. suffruticosa* roots are rich in gallotannins. MALDI‐MSI was first employed to analyze tissue‐specific distribution patterns of gallotannins in root cross‐sections, which could provide vital spatial information for the investigation of their biosynthetic pathways (Figure 1A). MSI results revealed the spatial distribution patterns of five gallotannins, including *β*G, digalloylglucose (DGG), trigalloylglucose (TGG), tetragalloylglucose (TeGG), and PGG, which show a significant difference between the xylem (wood core) and tissues outside of the xylem (root bark) (Figure 1B). *β*G, an acyl donor involved in gallotannin synthesis, is predominantly accumulated in the root bark. The spatial distributions of DGG and TGG are similar, mainly localized in the cork layer and phloem region of the root bark. MALDI‐MSI results indicated that these metabolites might accumulate in the phloem or be converted into more complex gallotannins through further enzymatic reactions. In contrast, the TeGG and PGG showed broad distribution, which was distributed in both the wood core and root bark, but mainly concentrated in the phloem region. Therefore, the significant differences in functions and metabolic activities between the wood core and root bark may be due to differential gene expression of gallotannin biosynthetic enzymes and could trigger regional regulation of the biosynthetic pathway.

### 2.2 Identification of UGTs Responsible for the Biosynthesis of *β*G

**FIGURE 2:**
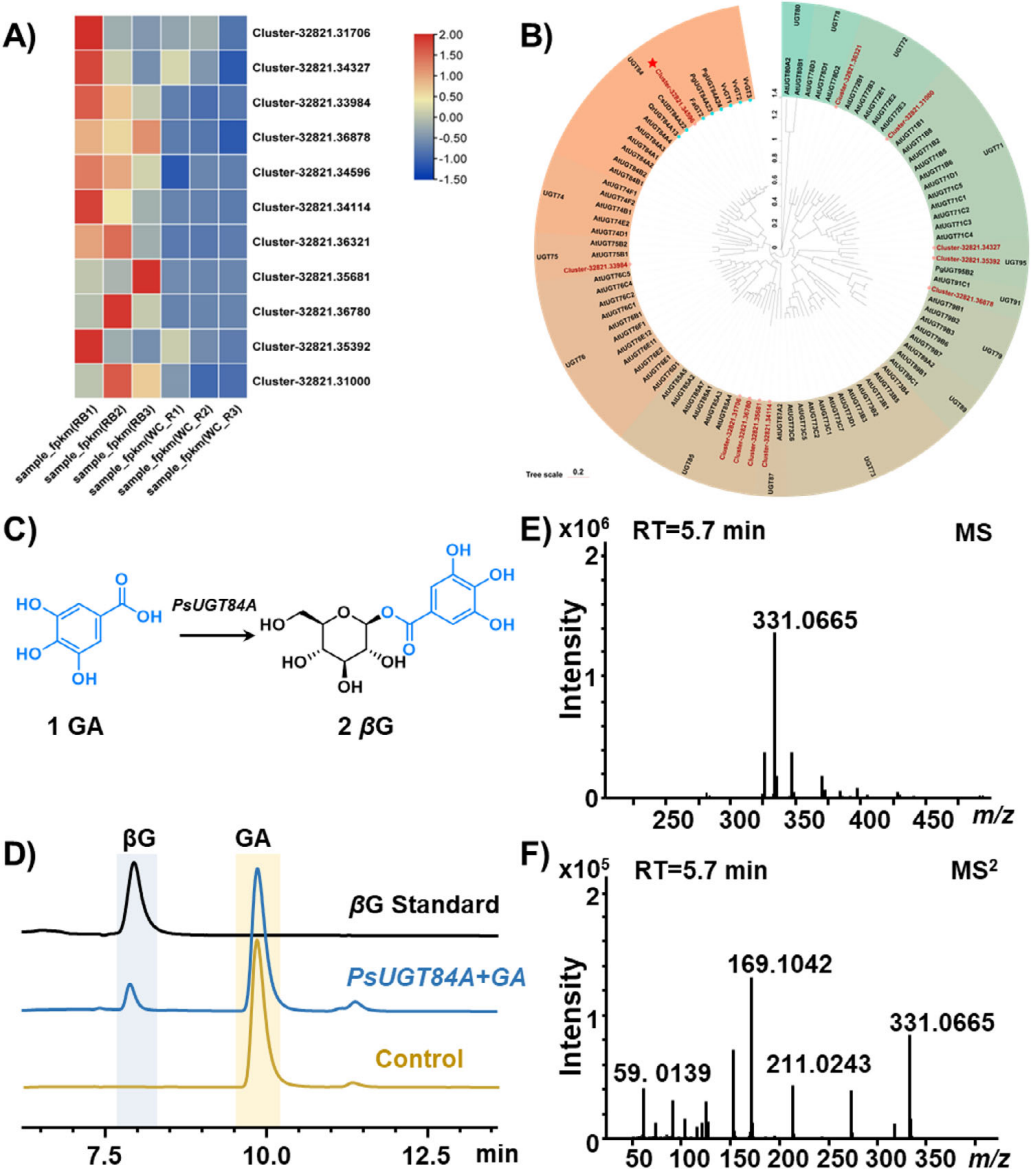
Functional identification of UGT genes. (A) Gene expression heatmap of *PsUGTs* in root bark (RB) and wood core (WC). (B) Phylogenetic analysis of selected UGTs from *P. suffruticos* *a*, *A. thaliana*, and other known UGTs. The clades were annotated based on the AtUGT family classification. The detailed list is available in Table S1, and an enlarged Figure 2B is available in the SI. (C) Diagram of UGT catalyzing gallic acid (GA) and UDP‐glucose to form *β*G. (D) HPLC for the determination of enzymatic reactions. (E) MS and (F) MS/MS spectra of the product 2.

As shown in Figure 1B, MALDI‐MSI results imply that the UDP‐glycosyltransferase (UGT) involved in *β*G biosynthesis might be expressed at a higher level in the root phloem. Guided by this spatial accumulation pattern, tissue‐specific transcriptomic sequencing (GenBank accession numbers: PRJNA1393498) was performed to identify candidate UGT genes. As a result, 11 significantly upregulated genes among the 94 annotated UGTs were identified according to the regional transcriptome, which held with high probability as functional genes involved in *β*G glycosylation (Figure 2A). Then, the 11 *PsUGTs* were subjected to phylogenetic tree analysis with *AtUGTs* from *Arabidopsis thaliana* and other plant UGTs, which were experimentally validated for transglycosylation activity on gallic acid [28]. Most *PsUGTs* were dispersed across different evolutionary branches in the phylogenetic tree, indicating their potential involvement in distinct metabolic pathways. Nevertheless, the gene 32821.34596 exhibits a unique cluster with members of the UGT84A subfamily in *A. thaliana* and other plants that catalyze the formation of 1‐galloylglucose from gallic acid in the same branch (Figure 2B). Therefore, this gene, named *PsUGT84A*, was selected as a candidate gene and expressed in *Escherichia coli* (Figure S1). Its catalytic activity was validated using gallic acid as the substrate and UDP‐glucose as the glycosyl donor. HPLC and LC‐MS/MS results showed that a new peak was generated, which was identified as *β*G ([M‐H]^−^, *m/z* 331.0665) by comparison with the standard (Figure 2C–E). The MS/MS spectra of the product showed characteristic fragment ions at *m/z* 211.02, *m/z* 169.10, and *m/z* 59.01, indicating the addition of one glucosyl moiety to gallic acid (Figure 2F). *PsUGT84A* exhibited the highest catalytic efficiency under the reaction conditions of acetic acid‐sodium acetate buffer (pH 5.5) at 35°C, and its activity was independent of metal ions (Figure S2). Therefore, a glycosyltransferase, PsUGT84A, was identified for the first time from *P. suffruticos* *a* that is capable of catalyzing the glycosylation of gallic acid to produce *β*G.

### 2.3 Identification of SCPLs Responsible for the Biosynthesis of Gallotannins

**FIGURE 3:**
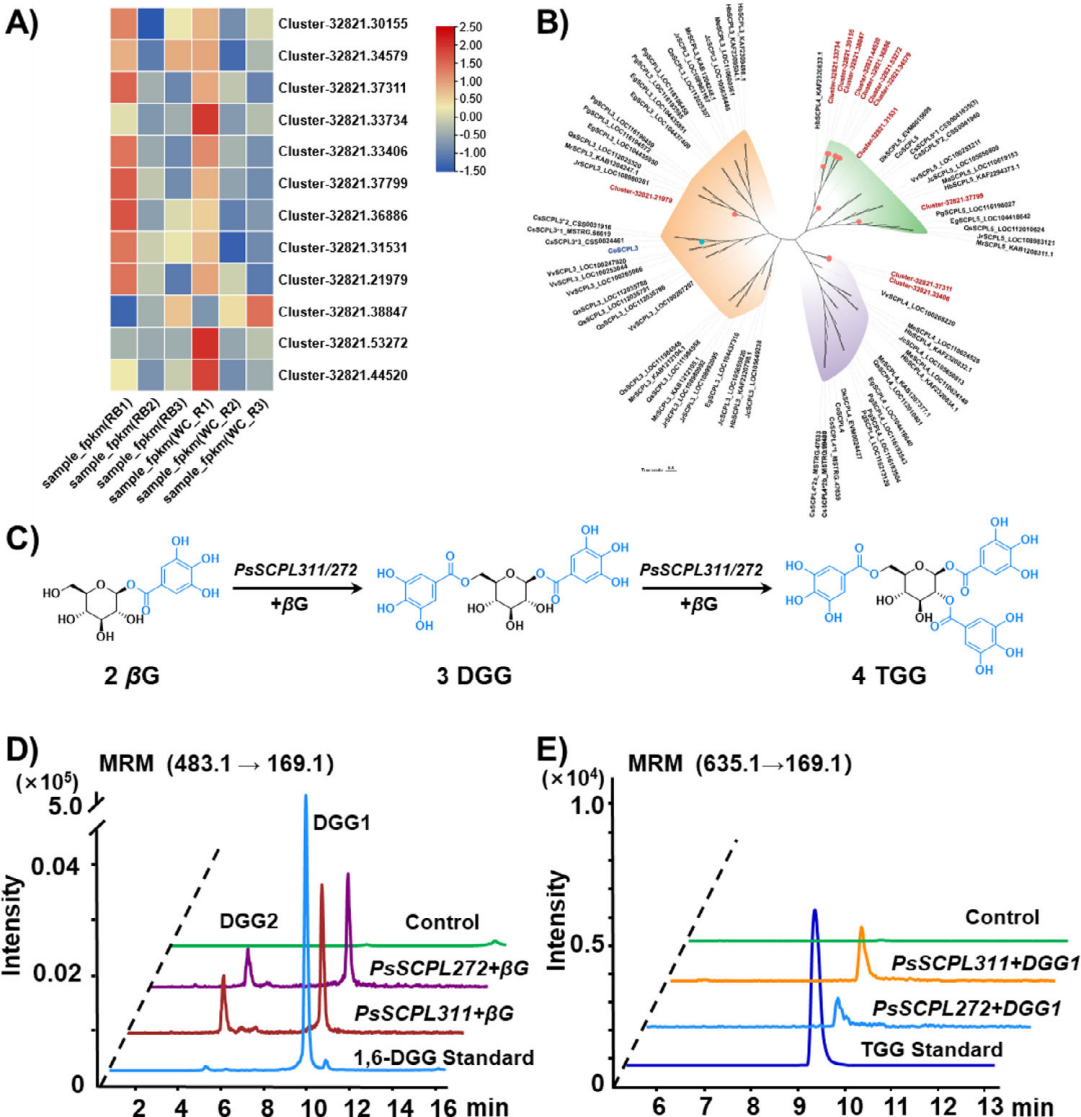
Functional identification of two *PsSCPLs*. (A) Gene expression heatmap of *PsSCPLs* in root bark (RB) and wood core (WC). (B) Phylogenetic analysis of selected SCPL‐AT homologs from 16 plant species. The detailed list is available in Table S2, and an enlarged Figure 3B is available in the SI. (C) Diagram of SCPL‐ATs catalyzing *β*G to form DGG and TGG. (D,E) LC‐MS/MS for the determination of the enzymatic activity of PsSCPL272 and PsSCPL311 using *β*G as the substrate.

According to the proposed biosynthetic pathway, subsequent steps involve sequential acylation of the 6‐OH, 2‐OH, 3‐OH, and 4‐OH at the central glucose molecule of *β*G to generate DGG, TGG, TeGG, and PGG. According to the functional annotations and the differential gene expression levels between the root bark and wood core, 12 candidate SCPL‐AT genes were efficiently mined from 75 SCPL genes (Figure 3A). A phylogenetic tree was constructed using these 12 genes together with validated SCPL family genes from other species (Figure 3B). The phylogenetic branches were classified into three groups: SCPL3, SCPL4, and SCPL5. Among them, one *PsSCPL* belonged to the SCPL3 clade, two *PsSCPLs* to the SCPL4 clade, and nine *PsSCPLs* to the SCPL5 clade. Previous studies on galloylation of tannins in tea and strawberry have shown that enzymes in the SCPL3 and SCPL4 clades directly participate in the transfer of the galloyl group, while the SCPL5 clade may act as scaffold proteins to assist their synthesis [21, 22, 29]. However, the functional conservation across species remains unclear. Considering the differences in genetic distance between the Ranunculaceae family and the reported species (Theaceae and Rosaceae), the screening threshold was appropriately relaxed to avoid missing potential functional genes, and all 12 candidate genes were subject to functional validation.

The coding sequences of candidate *PsSCPLs* were cloned into the pYES2 vector and heterologously expressed in *Saccharomyces cerevisiae* WTA11 cells. Following incubation of yeast microsomes with *β*G, MgCl_2_, and ATP, the precursor→product ion pairs of *m/z* 483.1→169.1 and *m/z* 635.1→169.1 were detected by LC‐MS/MS in the experimental groups of *PsSCPL272* and *PsSCPL311*, respectively. In Figure 3D, the major peaks were assigned to 1,6‐DGG (DGG1) by comparison of retention time, precursor ion, and fragment ions with the reference standard. In addition, a minor peak at retention time of 4.5 min was putatively identified as an isomer of 1,6‐DGG (DGG2). Due to the low yield of catalytic products, it is difficult to enrich enough amounts for NMR analysis. Similarly, in Figure 3E, the major peaks were assigned to 1,2,6‐TGG, and no other isomers were detected. Therefore, both PsSCPL272 and PsSCPL311 could catalyze the sequential acylation of *β*G to form DGG and TGG. Additionally, when *β*G served as the acyl donor and 1,6‐DGG as the acyl acceptor, the formation of TGG was also observed, indicating that *PsSCPL272* and *PsSCPL311* could also directly participate in the acylation of DGG (Figure S3).

**FIGURE 4:**
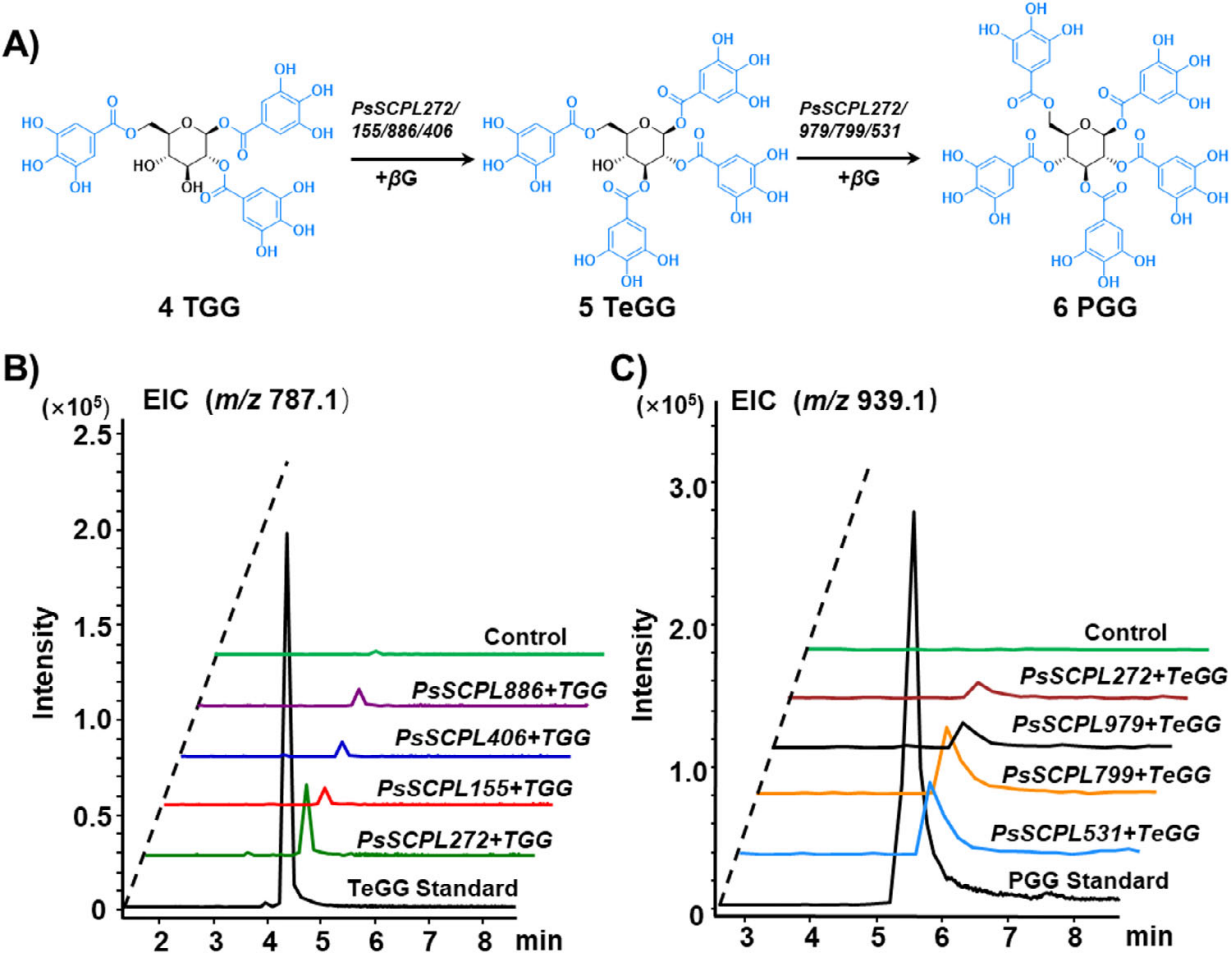
Functional identification of seven *PsSCPLs*. (A) Diagram of SCPL‐ATs catalyzing TGG to form TeGG and PGG. (B) LC‐MS for the determination of the enzymatic activity of PsSCPL272, PsSCPL155, PsSCPL406, and PsSCPL886 using *β*G and TGG as the substrate. (C) LC‐MS for the determination of the enzymatic activity of PsSCPL531, PsSCPL799, PsSCPL979, and PsSCPL272 using *β*G and TeGG as the substrate.

When using *β*G as the acyl donor and TGG as the acyl acceptor (Figure 4A), the reactions catalyzed by PsSCPL272, PsSCPL155, PsSCPL406, and PsSCPL886 yielded a base peak at *m/z* 787.1, which was assigned to TeGG by comparison of its tandem mass spectrum and retention time with standard reference (Figure 4B; Figure S4). Further experiments showed that when using TeGG as the acyl acceptor, PsSCPL531, PsSCPL799, PsSCPL979, and PsSCPL272 exhibited catalytic activity to yield a based peat at *m/z* 939.1, which was assigned to PGG by comparison of its tandem mass spectrum and retention time with reference standards (Figure 4C; Figure S5). Additionally, whether PsSCPLs could further elongate the galloyl chain to synthesize more complex hydrolysable tannins was investigated. However, no products corresponding to hexa‐ or higher galloyl glucoses were detected when *β*G was used as the acyl donor and PGG as the acyl acceptor. Herein, for the first time, we identified a series of *PsSCPL* genes in *P. suffruticosa* that are involved in the biosynthetic pathway of PGG.

In the Theaceae and Rosaceae families, enzymes from the SCPL3 clade have been shown to catalyze four consecutive reactions in the biosynthesis of simple gallotannins [21, 22]. However, our study reveals that the biosynthesis of gallotannins in *P. suffruticosa* (Ranunculaceae) is a multi‐enzyme‐driven process in which enzymes from SCPL3, SCPL4, and SCPL5 clades participate in the biosynthesis without the assistance of a scaffolding protein. This divergence may reflect lineage‐specific evolutionary trajectories, while the intrinsic catalytic activity of SCPL3 is likely maintained through conserved catalytic residues shared among SCPL enzymes across different plant families. In contrast, *P. suffruticosa* is evolutionarily distant from these families, and its SCPL family may have undergone functional divergence or sub‐functionalization during evolution, enabling multiple SCPL‐ATs from different clades to be capable of catalyzing reactions. Moreover, the differential distribution of gallotannins in the root of *P. suffruticosa* may result from the spatiotemporal specificity of expression of distinct SCPL gene family members. Consistent with the current findings, earlier enzymatic studies performed by the Gross group also observed inconsistencies in the molecular weights of enzymes purified from *Quercus robur* (Fagaceae), which could catalyze these four reaction steps [17, 18, 19, 20].

### 2.4 De Novo Biosynthesis of Pentagalloylglucose in *Nicotiana Benthamiana* Leaves

**FIGURE 5:**
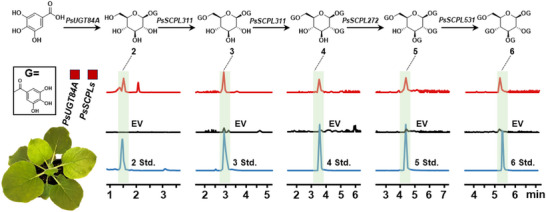
De novo biosynthesis of PGG in *Nicotiana benthamiana* leaves. Extracted ion chromatograms (EICs) of biosynthetic products. 2, *β*G; 3, DGG; 4, TGG; 5, TeGG; 6, PGG; EV, empty vector.

PGG is an important bioactive natural product; however, its extraction and purification are time‐consuming, labor‐intensive, and environmentally unfriendly. Therefore, engineering the biosynthetic pathways in chassis organisms is necessary. *N. benthamiana* is a biologically compatible and experimentally efficient platform for reconstructing the plant‐derived glycosylation‐acylation pathway. Therefore, all enzymes responsible for PGG biosynthesis were reconstructed in *N. benthamiana*. Four genes (*PsUGT84A*, *PsSCPL311*, *PsSCPL272*, and *PsSCPL531*) exhibiting relatively strong activity at each respective step of the PGG biosynthesis were selected to be co‐expressed in *N. benthamiana*. LC‐MS results showed that the final product, PGG, was successfully detected along with intermediates *β*G, DGG, TGG, and TeGG, achieving de novo synthesis of gallotannins from gallic acid in *N. benthamiana* for the first time (Figure 5).

## 3 Conclusions

In summary, an integrated screening strategy combining MALDI mass spectrometry imaging, spatial transcriptome sequencing, and phylogenetic analysis was developed for mining differentially expressed genes with high efficiency. Guided by the region‐specific distribution of gallotannins, we successfully discovered and identified nine key functional genes involved in gallotannins biosynthesis, including one UGT gene (*PsUGT84A*) and eight SCPL genes (*PsSCPL311*, *PsSCPL272*, *PsSCPL155*, *PsSCPL406*, *PsSCPL886*, *PsSCPL531*, *PsSCPL799*, and *PsSCPL979*) from *P. suffruticosa* roots. This strategy significantly narrows the candidate gene pool and markedly enhances gene discovery efficiency. Functional validation confirmed that these genes effectively drive the biosynthesis of pentagalloylglucose. This strategy not only provides essential enzymatic components for deciphering gallotannins biosynthesis but also offers a highly efficient solution for elucidating secondary metabolic pathways in non‐model plants.

## 4 Experimental Section

### 4.1 Plant Materials and Chemical Reagents

The roots of *Paeonia suffruticosa* Andr. were collected from the medicinal botanical garden of China Pharmaceutical University, China. The experimental reagents, including gallic acid (CAS: 149‐91‐7), UDP‐glucose (CAS: 133‐89‐1), *β*‐glucogallin (*β*G, CAS: 13405‐60‐2), 1,2,6‐trigalloylglucose (TGG, CAS: 79886‐49‐0), 1,2,3,6‐tetragalloylglucose (TeGG, CAS: 79886‐50‐3), and pentagalloylglucose (PGG, CAS: 14937‐32‐7) standards, were obtained from Yuan Ye Biology Company (Shanghai, China). The 1,6‐digalloylglucose (DGG, CAS: 23363‐08‐8) was synthesized in the laboratory.

### 4.2 MALDI MS Imaging

The 18 µm thickness of root sections was obtained at −20°C on the cryostat (Leica, Germany), and thaw‐mounted onto indium tin oxide (ITO)‐coated glass slides. A laboratory‐constructed electro‐sprayer was used to uniformly apply matrix solutions. Briefly, DHB (30 mg/mL, methanol/H_2_O, 7:3, V/V) was deposited on root sections for MALDI MSI in the positive mode. All measurements were performed using an ultrafleXtreme MALDI ToF/ToF mass spectrometer (Bruker Daltonics, USA) with a frequency tripled Nd:YAG solid state laser (λ = 355 nm). The laser was set to the “Ultra” footprint setting at a ∼100 µm diameter. MSI data was analyzed using flexAnalysis 3.4, DataAnalysis 4.0, and flexImaging 4.1 (Bruker Daltonics).

### 4.3 RNA Extraction and Transcriptome Sequencing

Total RNA from the root bark and wood core of *P. suffruticosa* root was isolated using the plant RNA isolation kit (Novogene, Beijing, China), respectively. RNA degradation and contamination were monitored on 1% agarose gel. The total RNA was reverse‐transcribed (RT) to cDNA with PrimeScript RT reagent kit (TaKaRa, Japan). RNA‐seq library preparation and sequencing were performed using the DNBSEQ platform (Beijing Genomics Institute). Gene expression was estimated by FPKM. The highly expressed genes in different regions of the *P. suffruticosa* root (FPKM≥20) were identified as candidate genes.

### 4.4 Phylogenetic Analyses

A phylogenetic tree was constructed using the neighbor‐joining method in MEGA11. Bootstrapping with 1000 replicates was performed to evaluate the reliability of the nodes. The detailed list is available in Tables S1 and S2.

### 4.5 Heterologous Expression of *PsUGT84A* in *E. coli* and Activity Assay

The candidate *PsUGT84A* gene cloned from *P. suffruticosa* was inserted into the pET28a vector at the Nde I and EcoRI restriction sites. The primers for molecular cloning of *PsUGT84A* are available in Table S3. The successfully sequenced plasmid was transformed into *E. coli* BL21 (DE3) competent cell used for protein expression at 18°C with 400 µm IPTG. After 12 h of induction, the bacterial culture was harvested, and the pellet was resuspended in buffer A (20 mm HEPES, pH 7.5, 500 mm NaCl, and 20 mm imidazole, pH 7.5). Then, ultrasonic treatment was used to lyse the bacterial cells, followed by centrifugation at 25 000 rpm for 1 h to obtain the supernatant. The crude PsUGT84A protein was purified with a Ni‐NTA affinity column (Smart‐Lifesciences, China), and the target protein was eluted with buffer B (20 mm HEPES, 500 mm NaCl, 300 mm imidazole, 10% (V/V) glycerol, pH 7.5). The concentrated protein was analyzed by SDS‐PAGE and Nanodrop. For the PsUGT84A activity test, the enzymatic reaction system (100 µL) contained 50 mm buffer solution, 40 µg purified proteins, 200 µm gallic acid, and 2 mm UDP‐glucose. Reactions were quenched with ice‐cold MeOH and centrifuged at 12 000 rpm for 10 min, and the supernatant was analyzed by HPLC and LC‐MS.

The biochemical properties of PsUGT84A were next investigated. To test the optimum pH value for PsUGT84A activity, assays were performed in different reaction buffers ranging in pH values from 4.0–6.0 (acetic acid‐sodium acetate buffer), 6.0–8.0 (Na_2_HPO_4_‐NaH_2_PO_4_ buffer), and 7.0–9.0 (Tirs‐HCl buffer). To study the optimum reaction temperature for PsUGT84A activity, reactions were incubated at different temperatures (10, 15, 25, 30, 35, 40, 45, and 50°C). To determine the necessity of divalent metal ions for PsUGT84A activities, Ca^2+^, Mg^2+^, Co^2+^, Fe^3+^, Fe^2+^, Cu^2+^, Mn^2+^, Ni^2+^, and Ag^+^ were used individually at a final concentration of 5 mm. Kinetic parameters of PsUGT84A for gallic acid were determined under the optimum reaction conditions. The assay was repeated three times, and Vmax and Km values were obtained by nonlinear fitting of the Michaelis‐Menten equation to the assay data.

### 4.6 Heterologous Expression of *PsSCPLs* in *S. cerevisiaein* and Activity Assay

The candidate *PsSCPL* genes cloned from *P. suffruticosa* were inserted into the pYES2‐Ura vector at BamHI and XhoI restriction sites. The primers used for molecular cloning of *PsSCPLs* are available in Table S4. The pYES2‐Ura vector carrying the *PsSCPLs* genes was transformed into *S. cerevisiae* strain WAT11 and cultured in SD medium at 30°C until OD_600_ reached 0.8–1.0. The cells were then collected and washed with sterile water. Protein expression was induced using an SG medium containing galactose after washing the cells three times. When the culture reached appropriate density, yeast cells were harvested by centrifugation and resuspended in 5 mL TEK buffer (50 mm Tris‐HCl, 1 mm EDTA, 100 mm KCl, pH 7.4), followed by 5 min incubation at room temperature. After centrifugation (12 000 rpm), the pellet was resuspended in 40 mL TESB buffer (50 mm Tris‐HCl, 1 mm EDTA, 0.6 m D‐sorbitol, pH 7.4). Cell lysis was performed using a high‐pressure homogenizer, and the lysate was centrifuged at 12 000 rpm for 10 min at 4°C to collect the supernatant. Yeast microsomes were precipitated by adding 0.15 m NaCl and 0.1 g/mL PEG‐4000 to the supernatant, followed by 15 min incubation on ice. The microsomal fraction was pelleted through centrifugation (12 000 rpm, 1 h) and resuspended in TEG buffer (50 mm Tris‐HCl, 1 mm EDTA, 20% glycerol, and pH 7.4). Protein concentration and purity were measured using an Eppendorf BioPhotometer, with final concentrations adjusted to 20–40 mg/mL. Samples were either used immediately for assays of enzymatic activity or stored at −80°C.

The enzymatic assay was conducted in a 100 µL reaction mixture, comprising 50 mM PBS buffer (pH 7.4), 0.4 mm *β*G (galloyl donor), 0.4 mm DGG, TGG, or TeGG (galloyl acceptor), 2 mm ascorbic acid, and 100 µg of yeast microsomes for incubation at 35°C for 3 h. After that, the reaction was stopped by adding 100 mL of methanol. The mixture was centrifuged at 12 000 rpm for 10 min, and the supernatant was collected. The precipitate was sonicated with 100 µL of methanol for 1 h. The mixed supernatant was filtered with a 0.22 µm PES filter before LC‐MS analysis.

### 4.7 Transient Expression of Candidate Genes in *N. benthamiana*

The candidate genes were introduced into the pEAQ‐HT vector at the AgeI and XhoI restriction sites. The primer pairs used for vector construction are listed in Table S5. The correctly sequenced recombinant plasmids were then transformed into the *Agrobacterium* strain GV3101 using a freeze‐thaw method. The positive transformants were subsequently cultured in 20 mL of liquid LB medium containing 50 µg/mL kanamycin and 25 µg/mL rifampicin for 16 h at 28°C. The cells were subsequently centrifuged at 6000 rpm for 10 min, and the supernatant was removed. The cell pellet was resuspended in the infiltration buffer (10 mm MES, 10 mm MgCl_2_, and 150 µm acetosyringone). For testing individual strain, Agrobacterium suspensions were adjusted to an OD_600_ of 0.6. When strains were used in combination, the Agrobacterium suspensions for each strain were diluted to an OD_600_ of 0.4 and mixed in equal concentration. After incubation at room temperature for 2 h, a needleless syringe was used to infiltrate these bacterial suspensions into the underside of 4–5‐week‐old *N. benthamiana* leaves. At three days post‐infiltration, a 200 µm substrate solution in water was infiltrated into the infected area of the leaf. The substrate‐infiltrated leaves were collected three days later and extracted with methanol for LC‐MS analysis (n = 6).

### 4.8 HPLC and LC‐MS Analysis

A Shimadzu LC‐2010C system (Shimadzu, Japan) was used for HPLC analysis. Samples were separated on a Hedera ODS‐2 C18 column (250 mm × 4.6 mm, 5 µm). The mobile phase consisted of water containing 0.1% formic acid (V/V, A) and methanol (B). The flow rate was 0.5 mL/min, and the column temperature was 35°C. The detection wavelength was 280 nm. The gradient elution program for detecting enzyme catalytic products was as follows: 0–5 min, 30%–65% B; 5–12 min, 65%–95% B; 12–14 min, 95%–30% B; 14–18 min, 30% B. MS analysis was performed on Agilent 6545 LC‐Q‐TOF MS equipped with a heated ESI source (Agilent Technologies, USA) in the negative ion mode. The parameters were as follows: sheath gas temperature, 320°C; ion spray voltage, 3500 V; fragmentor, 175 V; skimmer, 65 V; octupole 1 RF Vpp, 750 V. For MS/MS analysis, 10, 20, and 40 V collision energies were used. MassHunter Qualitative Analysis software was used for data processing. In terms of LC‐MS/MS analysis of gallotannin isomers, the LC separation parameters were followed from previous work [22].

## Conflicts of Interest

The authors declare no conflicts of interest.

## Supporting information




**Supporting File**: advs74776‐sup‐0001‐SuppMat.docx

## Data Availability

The data that support the findings of this study are available in the supplementary material of this article.
