# Supplementary material for: Mass Spectrometry Imaging‐Assisted Discovery of Gallotannin Biosynthetic Genes in the Root of Paeonia suffruticosa
Source: Adv Sci (Weinh). 2026 Mar 15;13(29):e14010. doi: 10.1002/advs.202514010 (PMC13205667; doi:10.1002/advs.202514010)

**Supporting Information**

**Mass spectrometry imaging-assisted discovery of gallotannin biosynthetic genes in the root of Paeonia suffruticosa**

Yushi Liu^a,1^, Wenna Duan^a,1^, Weiwei Tang^a,^*, Yucheng Zhao^b,^*, Bin Li^a,^*

^a^ State Key Laboratory of Natural Medicines and School of Traditional Chinese Pharmacy, China Pharmaceutical University, Nanjing, China.

^b^ Department of Resources Science of Traditional Chinese Medicines, School of Traditional Chinese Pharmacy, China Pharmaceutical University, Nanjing, China

* Corresponding authors:

E-mail: weiweitang@cpu.edu.cn (W. Tang), zhaoyucheng1986@126.com (Y. Zhao), binli@cpu.edu.cn (B. Li)

^1^ These authors made equal contributions to this work.

**Table S1.** Templates for transcriptome screening of candidate UGTs

| Name | Organism | GenBank ID |
| --- | --- | --- |
| AtUGT80A2 | *A. thaliana* | NM_180198 |
| AtUGT80B1 | *A. thaliana* | NM_103487 |
| AtUGT91C1 | *A. thaliana* | NM124347 |
| AtUGT79B1 | *A. thaliana* | NM_124785 |
| AtUGT79B6 | *A. thaliana* | NM_124780 |
| AtUGT79B7 | *A. thaliana* | NM_202795 |
| AtUGT79B2 | *A. thaliana* | NM_118891 |
| AtUGT79B3 | *A. thaliana* | NM_118892 |
| AtUGT89A2 | *A. thaliana* | NM_120429 |
| AtUGT89B1 | *A. thaliana* | NM_106048 |
| AtUGT89C1 | *A. thaliana* | NM_100480 |
| AtUGT73B4 | *A. thaliana* | NM_127109 |
| AtUGT73B5 | *A. thaliana* | NM_001202599 |
| AtUGT73B1 | *A. thaliana* | NM_119576 |
| AtUGT73B2 | *A. thaliana* | NM_119575 |
| AtUGT73B3 | *A. thaliana* | NM_119574 |
| AtUGT73D1 | *A. thaliana* | NM_115175 |
| AtUGT73C7 | *A. thaliana* | NM_115176 |
| AtUGT73C2 | *A. thaliana* | NM_129231 |
| AtUGT73C1 | *A. thaliana* | NM_129230 |
| AtUGT73C5 | *A. thaliana* | NM_129235 |
| AtUGT73C6 | *A. thaliana* | NM_129234 |
| AtUGT72B1 | *A. thaliana* | NM116337 |
| AtUGT72B3 | *A. thaliana* | NM_001331274 |
| AtUGT72E1 | *A. thaliana* | NM_114934 |
| AtUGT72E2 | *A. thaliana* | NM_126067 |
| AtUGT72E3 | *A. thaliana* | NM_122532 |
| AtUGT71B1 | *A. thaliana* | NM_113070 |
| AtUGT71B2 | *A. thaliana* | NM_113071 |
| AtUGT71B8 | *A. thaliana* | NM_113075 |
| AtUGT71B5 | *A. thaliana* | NM_001341025 |
| AtUGT71B6 | *A. thaliana* | NM_113073 |
| AtUGT71D1 | *A. thaliana* | NM_128527 |
| AtUGT71C5 | *A. thaliana* | NM_100598 |
| AtUGT71C1 | *A. thaliana* | NM_128529 |
| AtUGT71C2 | *A. thaliana* | NM_128528 |
| AtUGT71C3 | *A. thaliana* | NM_100600 |
| AtUGT71C4 | *A. thaliana* | NM_100599 |
| AtUGT78D1 | *A. thaliana* | NM_102790 |
| AtUGT78D2 | *A. thaliana* | NM_121711 |
| AtUGT78D3 | *A. thaliana* | NM_121709 |
| AtUGT85A4 | *A. thaliana* | NM_106476 |
| AtUGT85A5 | *A. thaliana* | NM_202156 |
| AtUGT85A7 | *A. thaliana* | NM_102085 |
| AtUGT85A1 | *A. thaliana* | NM_102089 |
| AtUGT85A2 | *A. thaliana* | NM_102086 |
| AtUGT85A3 | *A. thaliana* | NM_102088 |
| AtUGT76D1 | *A. thaliana* | NM_128205 |
| AtUGT76E1 | *A. thaliana* | NM_125350 |
| AtUGT76E2 | *A. thaliana* | NM_12535 |
| AtUGT76E11 | *A. thaliana* | NM_114534 |
| AtUGT76E12 | *A. thaliana* | NM_114533 |
| AtUGT76F1 | *A. thaliana* | NM_115428 |
| AtUGT76B1 | *A. thaliana* | NM_111968 |
| AtUGT76C1 | *A. thaliana* | NM_120669 |
| AtUGT76C2 | *A. thaliana* | NM_120668 |
| AtUGT76C4 | *A. thaliana* | NM_120670 |
| AtUGT76C5 | *A. thaliana* | NM_120671 |
| AtUGT87A2 | *A. thaliana* | NM_128569 |
| AtUGT75B1 | *A. thaliana* | NM_001331554 |
| AtUGT75B2 | *A. thaliana* | NM_100432 |
| AtUGT74B1 | *A. thaliana* | NM_102256 |
| AtUGT74E2 | *A. thaliana* | NM_100448 |
| AtUGT74F1 | *A. thaliana* | NM_201953 |
| AtUGT74F2 | *A. thaliana* | NM_129944 |
| AtUGT74D1 | *A. thaliana* | NM_001336347 |
| AtUGT84B1 | *A. thaliana* | NM_127890 |
| AtUGT84B2 | *A. thaliana* | NM_127889 |
| AtUGT84A1 | *A. thaliana* | NM_117638 |
| AtUGT84A2 | *A. thaliana* | NM_113051 |
| AtUGT84A3 | *A. thaliana* | NM_117639 |
| AtUGT84A4 | *A. thaliana* | NM_117640 |
| CsUDT84A22 | *Camellia sinensis* | KP682362 |
| QrUGT84A13 | *Quercus robur* | NM_001426822 |
| FaGT2 | *Fragaria ananassa* | AY663785 |
| PgUGT84A23 | *Punica granatum* | NM_001426721 |
| PgUGT84A24 | *Punica granatum* | NM_001426723 |
| PgUGT95B2 | *Punica granatum* | MH507175 |
| VvGT1 | *Vitis vinifera* | JN164679 |
| VvGT2 | *Vitis vinifera* | JN164680 |
| VvGT3 | *Vitis vinifera* | NM_001280920 |

**Table S2.** Templates for transcriptome screening of candidate UGTs

| Name | Organism | GenBank ID |
| --- | --- | --- |
| DkSCPL5 | *Diospyros kaki* | AB195285 |
| CoSCPL5 | *Camellia oleifera* | XM_028223562 |
| CsSCPL5*1 CSS0041635(3) | *Camellia sinensis* | XM_028223556 |
| CsSCPL5*2_CSS0041940 | *Camellia sinensis* | XM_028223562 |
| VvSCPL5_LOC100253211 | *Vitis vinifera* | XM_002269403 |
| JcSCPL5_LOC105650809 | *Jatropha curcas* | XM_012237767 |
| MeSCPL5_LOC110619153 | *Manihot esculenta* | XM_021762416 |
| HbSCPL5_KAF2294373.1 | *Hevea brasiliensis* | XM_058151386 |
| PgSCPL5_LOC116196027 | *Punica granatum* | XM_031525534 |
| EgSCPL5_LOC104418642 | [*Eucalyptus grandis*](https://www.ncbi.nlm.nih.gov/Taxonomy/Browser/wwwtax.cgi?id=71139) | XM_010030036 |
| QsSCPL5_LOC112010624 | *Quercus suber* | XM_024042973 |
| JrSCPL5_LOC108983121 | *Juglans regia* | XM_018954660 |
| MrSCPL5_KAB1208311.1 | *Morella rubra* | MZ462939 |
| VvSCPL4_LOC100265220 | *Vitis vinifera* | XM_002272080 |
| MeSCPL4_LOC110624528 | *Manihot esculenta* | XM_021769724 |
| HbSCPL4_KAF2320832.1 | *Hevea brasiliensis* | XM_021787310 |
| JcSCPL4_LOC105650813 | *Jatropha curcas* | XM_037638303 |
| MeSCPL4_LOC110624149 | *Manihot esculenta* | XM_043960593 |
| HbSCPL4_KAF2320834.1 | *Hevea brasiliensis* | XM_021787298 |
| MrSCPL4_KAB1207377.1 | *Morella rubra* | MZ462938 |
| QsSCPL4_LOC112010401 | *Quercus suber* | XM_024042744 |
| EgSCPL4_LOC104418640 | *Eucalyptus grandis* | XM_010030035 |
| PgSCPL4_LOC116193543 | *Punica granatum* | [XM_031522286](https://www.ncbi.nlm.nih.gov/nucleotide/XM_031522286.1?report=genbank&log$=nucltop&blast_rank=1&RID=4S57Y2MC013) |
| PgSCPL4_LOC116193504 | *Punica granatum* | XM_031522229 |
| PgSCPL4_LOC116213120 | [*Punica granatum*](https://www.ncbi.nlm.nih.gov/Taxonomy/Browser/wwwtax.cgi?id=22663) | XM_031547940 |
| DkSCPL4_EVM0024427 | [*Diospyros kaki*](https://www.ncbi.nlm.nih.gov/Taxonomy/Browser/wwwtax.cgi?id=35925) | [AB473186](https://www.ncbi.nlm.nih.gov/nucleotide/AB473186.1?report=genbank&log$=nucltop&blast_rank=2&RID=4S5DZREC013) |
| CoSCPL4 | [*Camellia oleifera*](https://www.ncbi.nlm.nih.gov/Taxonomy/Browser/wwwtax.cgi?id=385388) | [MZ462936](https://www.ncbi.nlm.nih.gov/nucleotide/MZ462936.1?report=genbank&log$=nucltop&blast_rank=1&RID=4S5T125Z013) |
| CsSCPL4*1_MSTRG.47639 | *Camellia sinensis* | XM_028240029 |
| CsSCPL4*2b_MSTRG.99480 | [*Camellia sinensis*](https://www.ncbi.nlm.nih.gov/Taxonomy/Browser/wwwtax.cgi?id=4442) | [XM_028223557](https://www.ncbi.nlm.nih.gov/nucleotide/XM_028223557.1?report=genbank&log$=nucltop&blast_rank=1&RID=4S6H9XY5013) |
| CsSCPL4*2a_MSTRG.47633 | *Camellia sinensis* | XM_028223557 |
| JcSCPL3_LOC105649238 | *Jatropha curcas* | XM_012235830 |
| HbSCPL3_KAF2320798.1 | *Hevea brasiliensis* | XM_058135075 |
| JcSCPL3_LOC105650820 | *Jatropha curcas* | XM_037638258 |
| EgSCPL3_LOC104437310 | *Eucalyptus grandis* | XM_010050240 |
| JrSCPL3_LOC108992095 | *Juglans regia* | XM_018966542 |
| JrSCPL3_LOC108980992 | *Juglans regia* | XM_018952061 |
| MrSCPL3_KAB1212105.1 | *Morella rubra* | KAB1212105 |
| QsSCPL3_LOC111984558 | *Quercus suber* | XM_065777188 |
| MrSCPL3_KAB1212104.1 | *Morella rubra* | KAB1212104 |
| QsSCPL3_LOC111984548 | *Quercus suber* | XM_065777188 |
| VvSCPL3_LOC100267207 | *Vitis vinifera* | XM_002265118 |
| QsSCPL3_LOC112035786 | *Quercus suber* | NW_019814425 |
| QsSCPL3_LOC112035791 | *Quercus suber* | NW_019814426 |
| QsSCPL3_LOC112035788 | *Quercus suber* | NW_019814427 |
| VvSCPL3_LOC100265066 | *Vitis vinifera* | XM_010658543 |
| VvSCPL3_LOC100253044 | *Vitis vinifera* | XM_002268481 |
| VvSCPL3_LOC100247920 | *Vitis vinifera* | XM_003633151 |
| CoSCPL3 | *Camellia oleifera* | - |
| CsSCPL3*3_CSS0024461 | *Camellia sinensis* | XM_028228452 |
| CsSCPL3*1_MSTRG.56619 | *Camellia sinensis* | XM_028228453 |
| CsSCPL3*2_CSS0031916 | *Camellia sinensis* | XM_028228454 |
| JrSCPL3_LOC108980281 | *Juglans regia* | XM_035692293 |
| MrSCPL3_KAB1204247.1 | *Morella rubra* | KAB1204247 |
| QsSCPL3_LOC112025320 | *Quercus suber* | XM_024057972 |
| EgSCPL3_LOC104435950 | [*Eucalyptus grandis*](https://www.ncbi.nlm.nih.gov/Taxonomy/Browser/wwwtax.cgi?id=71139) | XM_010048671 |
| PgSCPL3_LOC116194572 | *Punica granatum* | XM_031523420 |
| PgSCPL3_LOC116196459 | *Punica granatum* | [XM_031526187](https://www.ncbi.nlm.nih.gov/nucleotide/XM_031526187.1?report=genbank&log$=nucltop&blast_rank=1&RID=4TR5R3SC013) |
| EgSCPL3_LOC104437409 | *Eucalyptus grandis* | [XM_010050350](https://www.ncbi.nlm.nih.gov/nucleotide/XM_010050350.3?report=genbank&log$=nucltop&blast_rank=1&RID=4TRKGT9U013) |
| EgSCPL3_LOC104435951 | *Eucalyptus grandis* | XM_010048673 |
| PgSCPL3_LOC116193595 | *Punica granatum* | XM_031522335 |
| PgSCPL3_LOC116196458 | *Punica granatum* | XM_031526186 |
| QsSCPL3_LOC112025307 | *Quercus suber* | XM_031099282 |
| JrSCPL3_LOC108983167 | *Juglans regia* | XM_018954724 |
| MrSCPL3_KAB1204248.1 | *Morella rubra* | KAB1204248 |
| JcSCPL3_LOC105636446 | *Juglans regia* | XM_012219719 |
| MeSCPL3_LOC110600561 | *Manihot esculenta* | XM_021737427 |
| HbSCPL3_KAF2309504.1 | *Hevea brasiliensis* | XM_058140091 |
| HbSCPL3_KAF2309498.1 | *Hevea brasiliensis* | XM_058141497 |

**Table S3.** Primers for molecular cloning of *PsUGT84A*

| Primers | Sequence 5'-3' |
| --- | --- |
| pET28a-*PsUGT84A*-F | GTGCCGCGCGGCAGCCATATGGTATCTGATCAAGCTTGC |
| pET28a-*PsUGT84A*-R | CGACGGAGCTCGAATTCCTAATTGTTGGACTTGTAGTTGC |

**Table S4.** Primers for molecular cloning of *PsSCPLs*

| Primers | Sequence 5'-3' |
| --- | --- |
| pYES2-*PsSCPL155-*F | CGAGCTCGGATCCATGGATTATTCTCTAAATACTGTTATTTTCC |
| pYES2-*PsSCPL155-*R | GATATCTGCAGAATTCTTAATGACTAGGCATGTGCTTG |
| pYES2-*PsSCPL272-*F | GTACCGAGCTCGGATCCATGGCTGCAACTTTCTCTC |
| pYES2-*PsSCPL272-*R | GATATCTGCAGAATTCTCACTTAGGAGCAACTCTTCC |
| pYES2-*PsSCPL311-*F | CGAGCTCGGATCCATGGCAGTCAAGTCCAC |
| pYES2-*PsSCPL311-*R | GGATATCTGCAGAATTCTCAAAACACTCCCTTCGC |
| pYES2-*PsSCPL406-*F | ACCGAGCTCGGATCCATGTATTACGAATACGGATGGAC |
| pYES2-*PsSCPL406-*R | ATATCTGCAGAATTCTTATACTGGATACCAATGAATCCACC |
| pYES2-*PsSCPL520-*F | GTACCGAGCTCGGATCCATGGAAAAATTCAACATTCTTGTTATC |
| pYES2-*PsSCPL520-*R | GGATATCTGCAGAATTCTTACTTGAGCTTCTCGACC |
| pYES2-*PsSCPL531-*F | GTACCGAGCTCGGATCCATGGCCATGAGCTATGTAATAC |
| pYES2-*PsSCPL531-*R | GATATCTGCAGAATTCTTAAATTGGTTTTCCTGCCAAC |
| pYES2-*PsSCPL579-*F | GTACCGAGCTCGGATCCATGGAAAATACAGTCTTTCTCTCTC |
| pYES2-*PsSCPL579-*R | GATATCTGCAGAATTCTCACATAGAAACAACCGAATTTTC |
| pYES2-*PsSCPL734-*F | GTACCGAGCTCGGATCCATGGAACCTAAGCCATGG |
| pYES2-*PsSCPL734-*R | GATATCTGCAGAATTCTCAAACTGCCCCTGGTAG |
| pYES2-*PsSCPL799-*F | GTACCGAGCTCGGATCCATGCAGACAATTGTCGAGAC |
| pYES2-*PsSCPL799-*R | GATATCTGCAGAATTCCTACTTTACAGTCGCATATGTCAG |
| pYES2-*PsSCPL847-*F | CCGAGCTCGGATCCATGCATAAATTTAAACGGGTTTTCC |
| pYES2-*PsSCPL847-*R | GATATCTGCAGAATTCTTAAGAGTCACTGACTTGTTCC |
| pYES2-*PsSCPL886-*F | ACCGAGCTCGGATCCATGGAAAAATTCAACATTCTTGTTATCTC |
| pYES2-*PsSCPL886-*R | ATATCTGCAGAATTCCTAGTATATTTCCTTCCGCGTC |
| pYES2-*PsSCPL979-*F | GTACCGAGCTCGGATCCATGACACTAGAAGCCACATC |
| pYES2-*PsSCPL979-*R | GATATCTGCAGAATTCTTAGAGGGGATAGTATGCAAGC |

**Table S5.** Primer for cloning active genes into plant expression vectors

| Primers | Sequence 5'-3' |
| --- | --- |
| pEAQ-*PsUGT84A-*F | AAATTCGCGACCGGTATGGTATCTGATCAAGCTTGC |
| pEAQ-*PsUGT84A-*R | GTTAAAGGCCTCGAGCTAATTGTTGGACTTGTAGTTGC |
| pEAQ-*PsSCPL272-*F | CCCAAATTCGCGACCGGTATGGCTGCAACTTTCTCTC |
| pEAQ-*PsSCPL272-*R | GTTAAAGGCCTCGAGTCACTTAGGAGCAACTCTTCC |
| pEAQ-PsSCPL311-F | CCCAAATTCGCGACCGGTATGGCAGTCAAGTCCAC |
| pEAQ-PsSCPL311-R | AGAGTTAAAGGCCTCGAGTCAAAACACTCCCTTCGCT |
| pEAQ-PsSCPL531-F | CCCAAATTCGCGACCGGTATGGCCATGAGCTATGTAATAC |
| pEAQ-PsSCPL531-R | AGAGTTAAAGGCCTCGAGTTAAATTGGTTTTCCTGCCAACC |

**
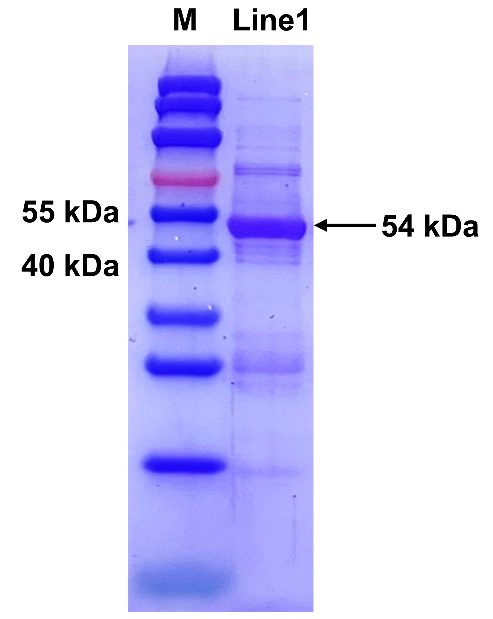
**

**Figure S1**. SDS-PAGE analysis of PsUGT84A24. M: Protein marker; Line 1: PsUGT84A pure enzyme.


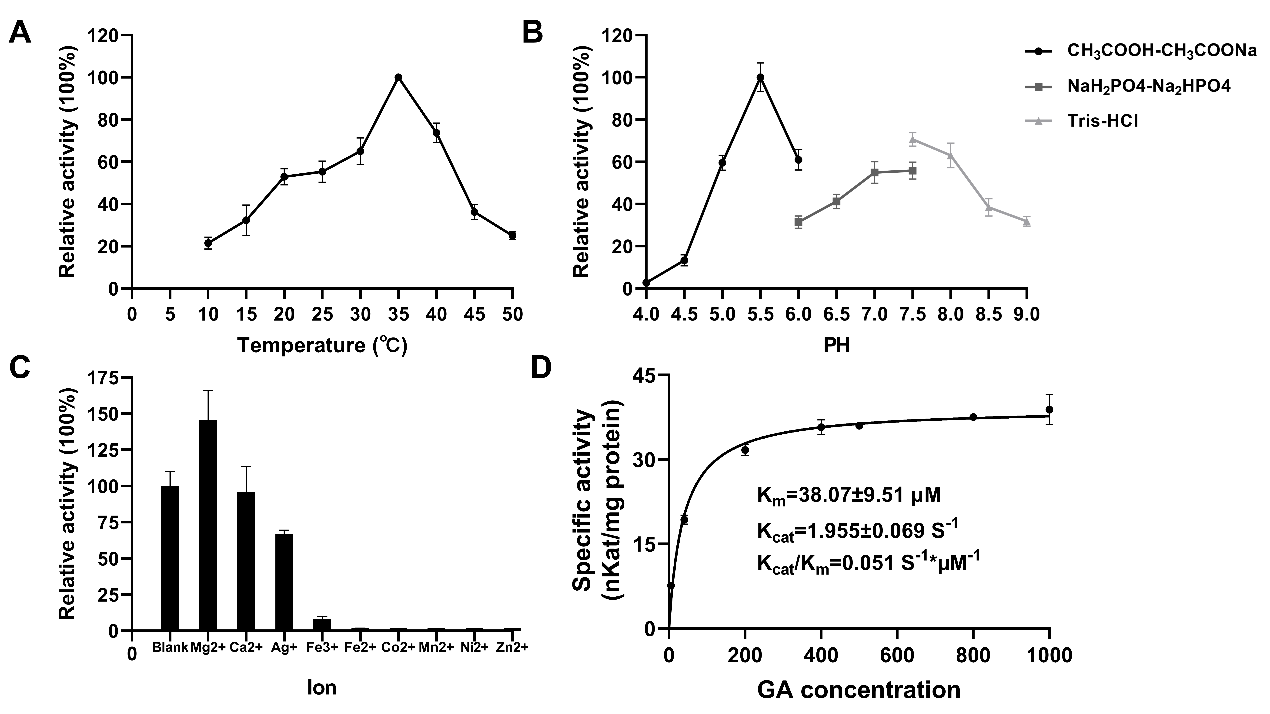


**Figure S2**. Study on the enzymatic properties of *PsUGT84A.* A) Effect of temperature on the activity of *PsUGT84*A. B) Effect of pH on the activity of *PsUGT84A*. C) Effect of metal ions on the activity of *PsUGT84A*. D) Kinetics of enzyme-catalyzed reactions


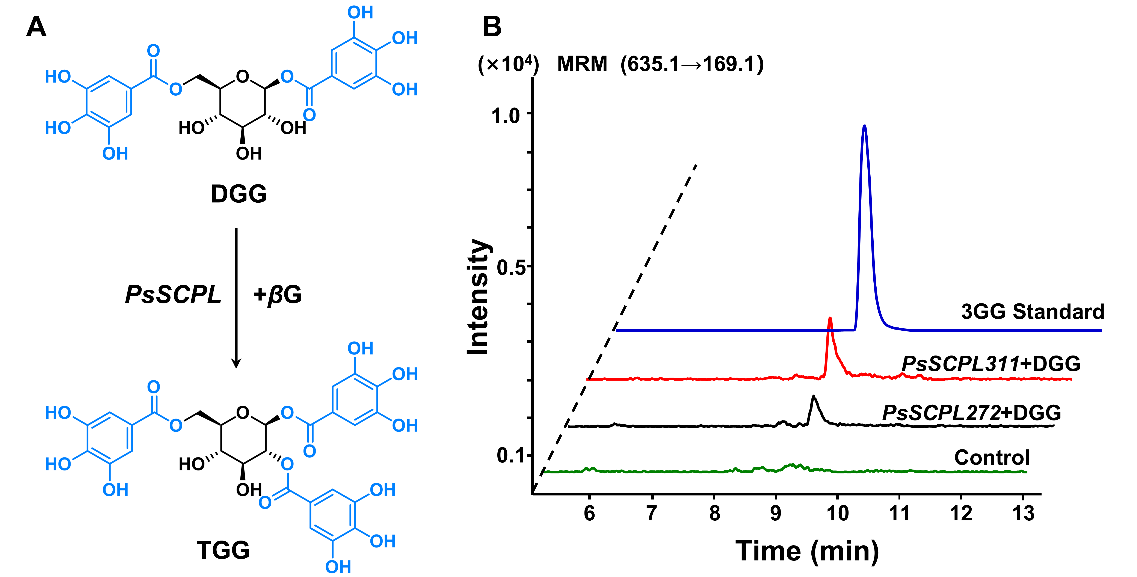


**Figure S3**. A) Catalytic reaction from DGG to TGG. B) Products of the activity of *PsSCPLs* with *β*G and DGG as substrates were identified using mass spectrometry.


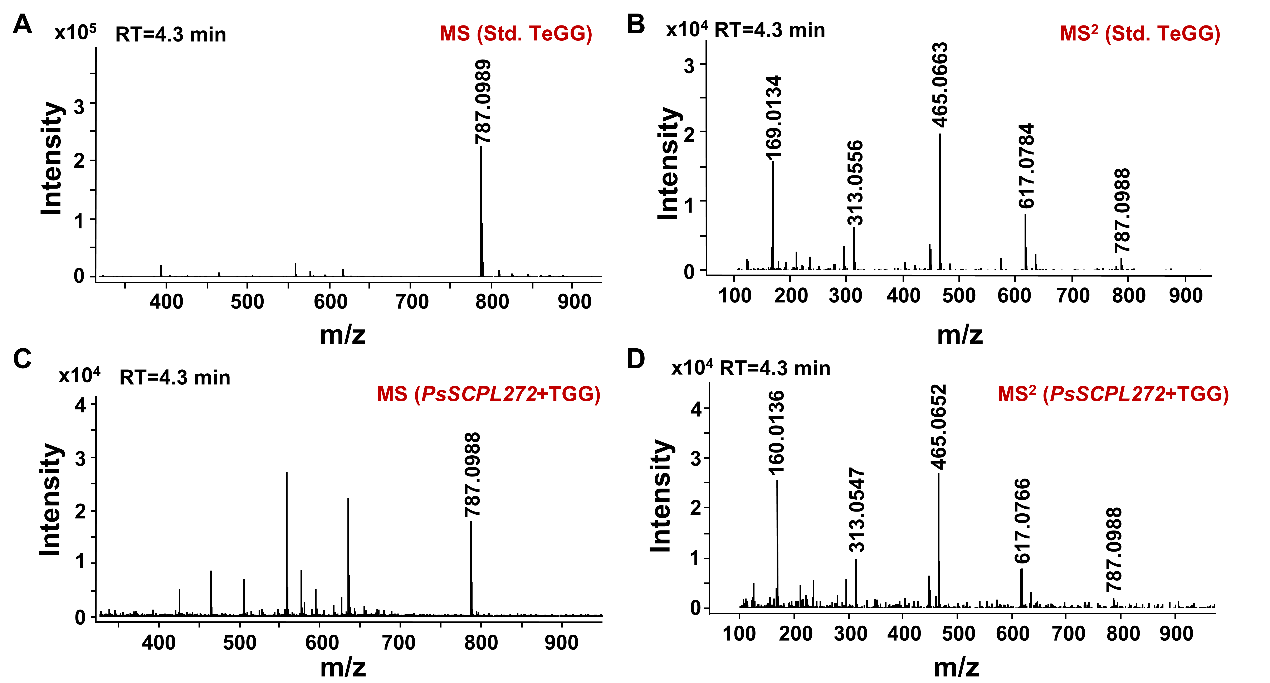


**Figure S4**. Mass spectra of enzymatic reaction products with *β*G and TGG as substrates. A) MS spectrum of TeGG standard. B) MS/MS spectrum of TeGG standard. C) MS spectrum of enzymatic reaction products catalyzed by *PsSCPL272*. D) MS/MS spectrum of enzymatic reaction products catalyzed by *PsSCPL272*.


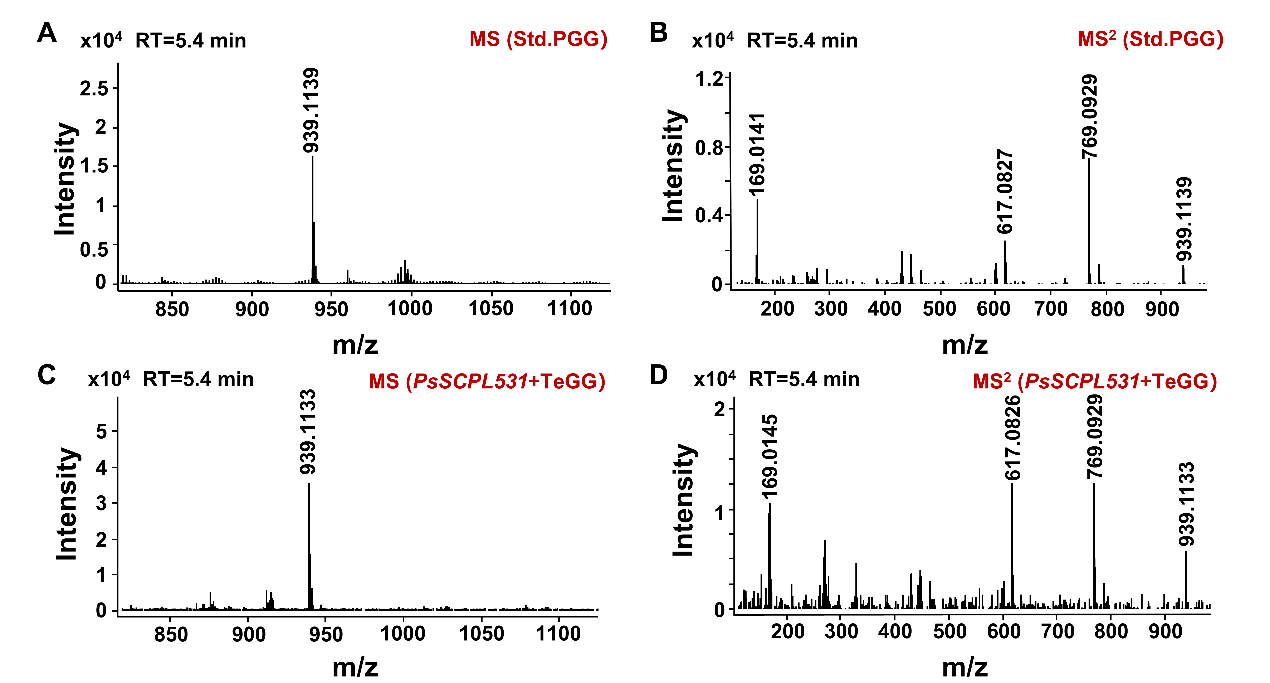


**Figure S5**. Mass spectra of enzymatic reaction products with *β*G and TeGG as substrates. A) MS spectrum of PGG standard. B) MS/MS spectrum of PGG standard. C) MS spectrum of enzymatic reaction products catalyzed by *PsSCPL531*. D) MS/MS spectrum of enzymatic reaction products catalyzed by *PsSCPL531*.

**Enlarged Figure 2B**


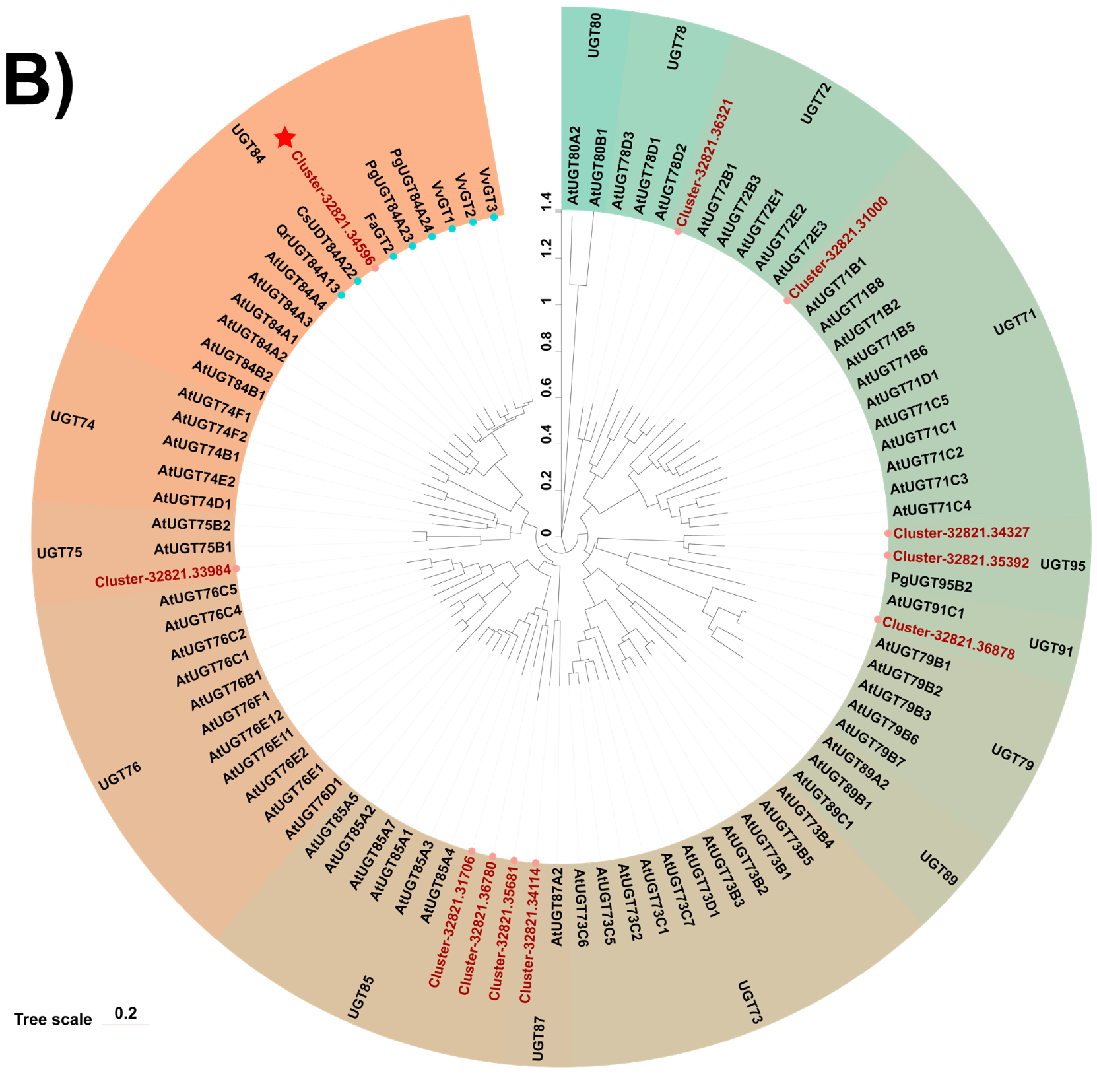


**Enlarged Figure 3B**


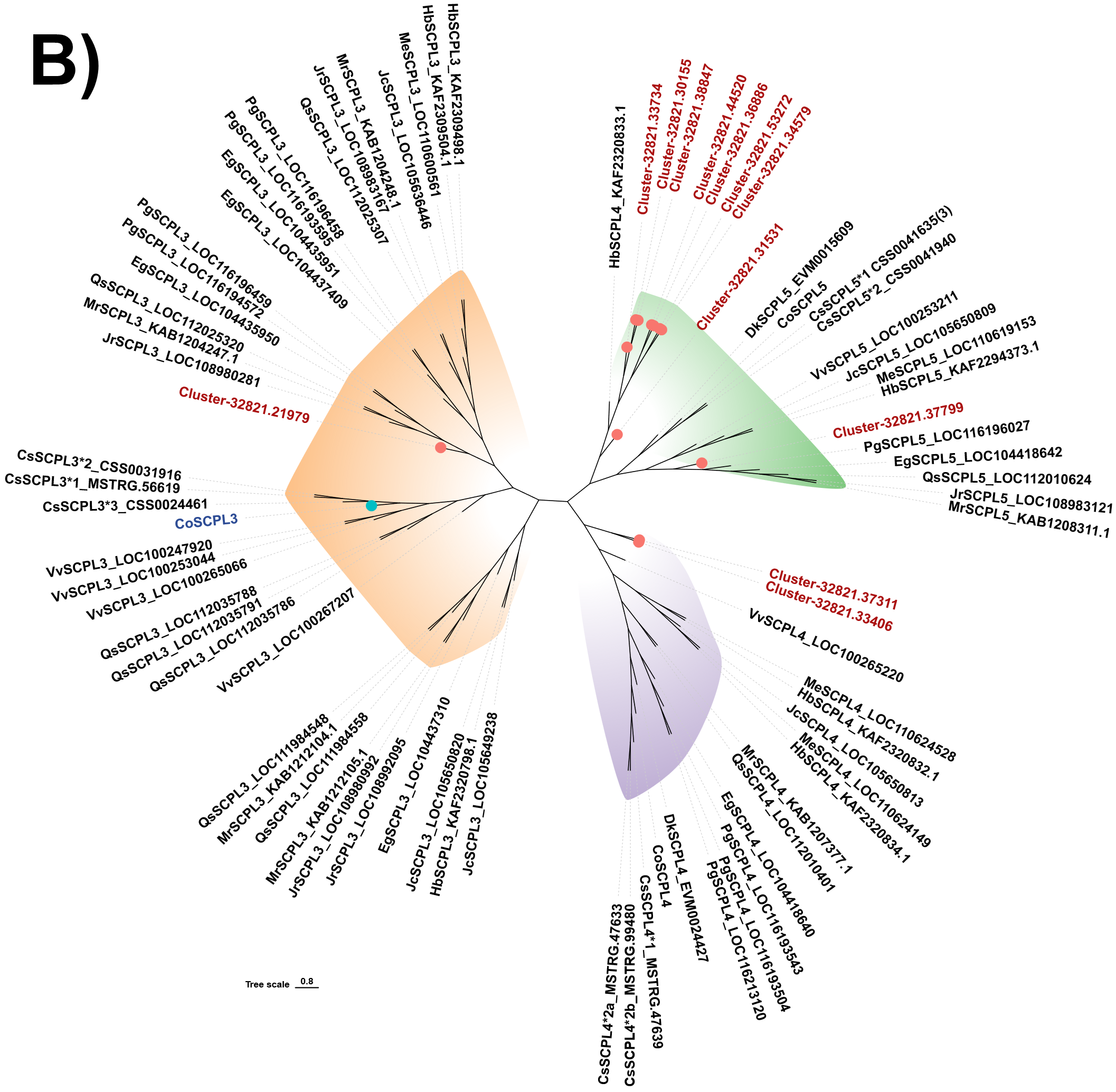

Supplement: Supplementary file 1 — Supporting File: advs74776‐sup‐0001‐SuppMat.docx [file ADVS-13-e14010-s001.docx]
